# Rural-Urban Differences in Cognitive Impairment and Medication Prescribing Pre- Dementia Diagnosis

**DOI:** 10.1093/geroni/igaf122.629

**Published:** 2025-12-31

**Authors:** Guneet Jasuja, Heather Davila, Haijuan Yan, Mark Zocchi, Michelle Hilgeman, Hilary Mosher, Tammy Walkner, Lauren Moo

**Affiliations:** Center for Health Optimization and Implementation Research, VA Bedford, Boston, Massachusetts, United States; Iowa City VA Healthcare System, Iowa City, Iowa, United States; Center for Health Optimization and Implementation Research, VA Bedford Healthcare System, Bedford, Massachusetts, United States; Center for Health Optimization and Implementation Research, VA Bedford Healthcare System, Bedford, Massachusetts, United States; Tuscaloosa VA Medical Center, Tuscaloosa, Alabama, United States; Minneapolis VA Medical Center, Minneapolis, Minnesota, United States; Iowa City VA Health Care System, Iowa City, Iowa, United States; VA Bedford Healthcare System, Bedford, Massachusetts, United States

## Abstract

Documentation of a dementia diagnosis provides access to a pathway of evidence-based treatment. However, delayed diagnosis is common, particularly in rural areas with limited access to dementia specialists, often resulting in documentation of less specific cognitive impairment and prescribing of dementia medications pre-dementia diagnosis. We described patterns of rural-urban differences in cognitive impairment and dementia medication prescribing pre-dementia diagnosis in the Veterans Affairs (VA), the largest integrated healthcare system in the U.S. We identified veterans ≥at least 50 years old with two ICD-10-CM dementia diagnoses, with first diagnosis in 2022 or 2023. Documentation of diagnoses of mild cognitive impairment (MCI), amnesia/memory loss, and receipt of dementia medications were compared among rural and urban veterans in the two years preceding the first dementia diagnosis. In 2022-23, 89,482 veterans had a first dementia diagnosis (mean age: 79 years, 97% men). Of these 89,482 veterans, 67% resided in urban vs. 33% in rural areas, with 43% living in the South. More urban vs. rural veterans had documentation of MCI (20% vs. 17.5%) pre-dementia diagnosis. As expected, more rural Veterans had documentation of an amnesia/memory loss (15% vs. 13%), received a dementia medication (12% vs. 10%), and received a dementia medication without documentation of MCI or amnesia/memory loss pre-dementia diagnosis (5.1% vs. 4.2%) (p < 0.01 for all comparisons above). Particularly for rural veterans, dementia-targeting treatment often began well before dementia diagnosis. Whether these patterns reflect delays in formally classifying cognitive impairment as dementia, gaps in access to specialists or other factors should be further explored.

